# Ecocardiografia Terapêutica

**DOI:** 10.36660/abc.20220014

**Published:** 2022-04-07

**Authors:** David Le Bihan

**Affiliations:** 1 Universidade de São Paulo Instituto do Coração São Paulo SP Brasil Universidade de São Paulo Instituto do Coração, São Paulo, SP – Brasil; 2 Grupo Fleury São Paulo SP Brasil Grupo Fleury, São Paulo, SP – Brasil

**Keywords:** Infarto Agudo do Miocárdio/fisiopatologia, Diagnóstico por Imagem/tendências, Ecocardiografia/métodos, Ecocardiografia/tendências, Reperfusão Miocárdica/métodos, Reperfusão Miocárdica/tendências

Desde a sua introdução por Edler e Hertz, a ecocardiografia se tornou o principal exame da propedêutica cardiológica. A sua evolução nos últimos 50 anos foi marcante, desde as imagens de modo A, modo M, modo bidimensional e Doppler, até as modernas reconstruções tridimensionais e os *softwares* para estudo da deformação miocárdica usados atualmente. Por esse motivo, a ecocardiografia é capaz de fornecer não apenas dados diagnósticos, mas também importantes informações prognósticas, em virtualmente todas as doenças do coração.^1^

Um dos principais avanços da ecocardiografia foi o reconhecimento de substâncias capazes de aumentar o sinal de ultrassom, para melhor visibilização das estruturas cardíacas, popularmente conhecidas como “contrastes ecocardiográficos”. Seu início data do final da década de 60, com a primeira experiência reportada sobre a utilização de solução salina agitada associada ao ultrassom cardíaco.^2^ Essa técnica (agitação de solução fisiológica para que fique aerada, permitindo aumento da reflexão do som) é utilizada até hoje para detecção de comunicações intra-cardíacas, mas apresenta como inconvenientes a pouca estabilidade intravascular da solução e o fato das bolhas de ar serem eliminadas do corpo durante a passagem do sangue pelos pulmões impedindo, assim, seu emprego para visualização de estruturas do coração esquerdo, quando a solução salina agitada é administrada em veia periférica.^3^

Entretanto, foi o surgimento de meios de contraste ecocardiográfico de segunda geração, de fabricação industrial, que consolidou o emprego dessas substâncias na prática ecocardiográfica. Esses compostos correspondem a micropartículas estabilizadas de gás, que uma vez dentro do meio intravascular não modificam a circulação sanguínea, por serem menores que as hemácias e os capilares. Além disso, possuem estabilidade, podendo ser injetadas em veia periférica, passando íntegras pela circulação pulmonar, permitindo não apenas a opacificação do ventrículo esquerdo, mas também a detecção da perfusão miocárdica.^4-6^ Essas características conferem aos meios de contraste ultrassonográfico alta segurança, associada à grande melhora da qualidade da imagem, aumentando a acurácia diagnóstica.^7^

A capacidade dos meios de contraste ecocardiográfico atuais de permitir a visibilização mais nítida da separação entre o sangue e o miocárdio adjacente ocorre por conta de uma propriedade dessas moléculas, que sofrem oscilação volumétrica ao serem submetidas à energia ultrassônica. Isso acentua a reflexão do som, por meio do retorno à fonte emissora das ondas sonoras em sua frequência fundamental e em suas frequências harmônicas. Por outro lado, pulsos de energia ultrassônica de alta intensidade aplicados a esses contrastes levam ao fenômeno de cavitação inercial, ou seja, violenta expansão e colapso dessas micropartículas, gerando oscilação de pressão local e fortes ondas de choque no meio intravascular. A cavitação inercial é a base física para o fenômeno de sonotrombólise, que corresponde à capacidade dos pulsos de alta energia ultrassônica, associados à presença do contraste ecocardiográfico, levarem à quebra e dissolução de trombos.^8^ Essa possibilidade foi primeiramente demonstrada em modelos animais.^9,10^ Posteriormente, foi demonstrado que a sonotrombólise não apenas ajudava a dissolução de trombos, mas também atuava no endotélio vascular e na microcirculação, com liberação de substâncias vasodilatadoras, incluindo o óxido nítrico.^11^ Esse conhecimento foi o alicerce para utilização dessa técnica em estudos clínicos.

O grupo do professor Mathias e seus colaboradores foi pioneiro no desenvolvimento de ensaios randomizados, para testar a utilização da sonotrombólise como tratamento adjuvante no infarto agudo do miocárdio- IAM.^12-14^ Esses trabalhos mostraram que utilizando equipamentos de ultrassom e meios de contraste ecocardiográfico comercialmente disponíveis, em pacientes com IAM com supra desnivelamento do segmento ST, randomizados para receber ou não sonotrombólise como tratamento adjuvante à angioplastia primária, foi possível diminuir o fenômeno de obstrução microvascular,^12^aumentar a taxa de recanalização dos vasos epicárdicos e diminuir o tamanho da área necrótica,^13^ bem como diminuir o remodelamento ventricular e melhorar a função miocárdica em longo prazo.^14^

Nesta edição dos Arquivos, uma nova peça de conhecimento foi introduzida por esse mesmo grupo a partir da publicação de Tavares et al.^15^ Utilizando um grupo de 100 pacientes randomizados 1:1 para angioplastia primária ou angioplastia e sonotrombólise, os autores demonstraram uma melhora, no grupo submetido à sonotrombólise, do índice ecocardiográfico de motilidade e da perfusão ventricular esquerda, em uma avaliação ecocardiográfica realizada 6 meses após o IAM. Esses dois índices ecocardiográficos usados como desfecho são sabidamente definidores de prognóstico em doença coronariana e sua melhora no grupo submetido à sonotrombólise reflete a capacidade do método de ajudar na dissolução dos trombos em vasos coronários epicárdicos, mas também de melhorar a microcirculação.^16,17^ Um importante aspecto que devemos ressaltar é que o trabalho de Tavares B. et al.^15^ apresenta a característica única de demonstrar a utilidade do uso de contraste ecocardiográfico em toda sua amplitude de funções: opacificação ventricular, perfusão miocárdica e, finalmente, terapêutica antitrombótica.

A sonotrombólise quebra um paradigma da cardiologia. Certamente, nem mesmo Edler e Hertz imaginaram a amplitude que atingiria a ecocardiografia, que agora deixa o escopo meramente diagnóstico, para se tornar um importante adjuvante terapêutico. Em um país que sofre intensamente o impacto da doença obstrutiva coronariana (IAM correspondeu à 7,06% do total de óbitos no ano de 2017)^18^ e em que as terapias de reperfusão conhecidamente eficazes (angioplastia e fibrinólise) ainda não estão disponíveis para grande parte da população em tempo adequado (cerca de um quarto dos pacientes com IAM com supra de ST chegam ao hospital com mais de 6 horas de dor),^19^ o surgimento de uma nova alternativa terapêutica adjuvante pode ter forte impacto na saúde da população. Seguramente, para isso muitas perguntas ainda precisam de respostas: qual o impacto da sonotrombólise sobre a mortalidade pós IAM? Existe benefício também para pacientes que realizaram apenas fibrinólise? Existe benefício para as síndromes coronarianas sem supra desnivelamento de segmento ST? A sonotrombólise pode ser iniciada imediatamente após a dor, ainda no serviço de transporte pré-hospitalar? Esse caminho de conhecimento será longo e levará alguns anos. No entanto, o trabalho de Tavares B.,^15^ publicado nesta edição dos Arquivos representa uma importante contribuição.
