# Supplementary material for: Ecocardiografia Terapêutica
Source: Arq Bras Cardiol. 2022 Apr 7;118(4):766–7. [Article in Portuguese] doi: 10.36660/abc.20220014 (PMC9007021; doi:10.36660/abc.20220014)
Supplement: Translation

# Translation: Therapeutic Echocardiography

**Authors:** David Le Bihan

**Affiliations:** 1 Brazil Universidade de São Paulo Instituto do Coração, São Paulo, SP – Brazil; 2 Brazil Grupo Fleury, São Paulo, SP – Brazil

**Keywords:** Myocardial Acute Infarction/physiopathology, Diagnostic Imaging/trends, Echocardiography/methods, Echocardiography/trends, Myocardial Reperfusion/methods, Myocardial Reperfusion/trends

Since its introduction by Edler and Hertz, echocardiography has become the main test of cardiologic propedeutic. Over the last 50 years, its evolution has been remarkable, from A-mode, M-mode, two-dimensional and Doppler images to modern three-dimensional reconstructions and softwares for studying myocardial deformation currently used. For this reason, echocardiography can provide diagnostic data and important prognostic information in virtually all heart diseases.^1^

One of the major advances in echocardiography was the recognition of substances capable of increasing the ultrasound signal for better visualization of cardiac structures, popularly known as “echocardiographic contrasts.” Its beginning dates back to the late 1960s, with the first experience reported on the use of agitated saline solution associated with cardiac ultrasound.^2^ This technique (agitation of a saline solution so that it is aerated, allowing an increase in sound reflection) is still used to detect intracardiac communications. Still, it has as drawbacks the poor intravascular stability of the solution and the fact that air bubbles are eliminated from the body during the passage of blood through the lungs, thus preventing its use for visualization of structures of the left heart when the agitated saline solution is administered in a peripheral vein.^3^

However, the emergence of industrially manufactured second-generation echocardiographic contrast media consolidated the use of these substances in echocardiographic practice. These compounds correspond to stabilized microparticles of gas, which, once inside the intravascular environment, do not change blood circulation, as they are smaller than red blood cells and capillaries. In addition, they are stable and can be injected into a peripheral vein, passing intact through the pulmonary circulation, allowing left ventricular opacification and the detection of myocardial perfusion.^4-6^ These characteristics give high security to ultrasound contrast media, associated with a great improvement in image quality, increasing diagnostic accuracy.^7^

The ability of current echocardiographic contrast media to allow clearer visualization of the separation between the blood and the adjacent myocardium is due to a property of these molecules, which undergo volumetric oscillation when subjected to ultrasonic energy. This fact accentuates the reflection of sound through the return to the emitting source of sound waves in their fundamental frequency and harmonic frequencies. On the other hand, high-intensityultrasonic energy pulses applied to these contrasts lead to inertial cavitation, that is, violent expansion and collapseof these microparticles, generating local pressure oscillationand strong shock waves in the intravascular environment. Inertial cavitation is the physical basis for the phenomenon of sonothrombolysis, which corresponds to the capacityof high-energy ultrasonic pulses, associated with the presence of echocardiographic contrast, to lead to thrombus dissolution.^8^ This possibility was first demonstrated in animal models.^9,10^ Subsequently, it was shown that sonothrombolysis helped dissolve thrombi and acted on the vascular endothelium and microcirculation, releasing vasodilating substances, including nitric oxide.^11^ This knowledge was the foundation for using this technique in clinical studies.

The group of Professor Mathias and his collaborators pioneered the development of randomized trials to test sonothrombolysis as an adjuvant treatment in acute myocardial infarction - AMI.^12-14^ These studies showed that it was possible to use commercially available ultrasound equipment and echocardiographic contrast media in patients with ST-segment elevation AMI randomized to receive or not sonothrombolysis, as an adjuvant treatment to primary angioplasty, to reduce the phenomenon of microvascular obstruction,^12^ increase the rate of recanalization of epicardial vessels and decrease the size of the necrotic area,^13^ as well as decrease ventricular remodeling and improve long-term myocardial function.^14^

In this edition of the *Arquivos*, a new piece of knowledge was introduced by this same group after the publication by Tavares B. et al.^15^ Using a group of 100 patients randomized 1:1 to primary angioplasty or angioplasty and sonothrombolysis, the authors demonstrated an improvement in the group undergoing sonothrombolysis in the echocardiographic index of motility and left ventricular perfusion in an echocardiographic evaluation performed 6 months after the AMI. These two echocardiographic indices used as endpoints are known to define the prognosis in coronary heart disease, and their improvement in the group undergoing sonothrombolysis reflects the method’s ability to help the dissolution of thrombi in epicardial coronary vessels, but also to improve microcirculation.^16,17^ An important aspect that we must emphasize is that the work by Tavares B. et al.^15^ has the unique characteristic of demonstrating the usefulness of using echocardiographic contrast in all its range of functions: ventricular opacification, myocardial perfusion and, finally, antithrombotic therapy.

Sonothrombolysis breaks a paradigm of cardiology. Certainly, not even Edler and Hertz imagined the scope that echocardiography would reach, which now leaves the merely diagnostic scope, to become an important therapeutic adjuvant. In a country that suffers intensely from the impact of obstructive coronary disease (AMI corresponded to 7.06% of total deaths in 2017)^18^ and where reperfusion therapies that are known to be effective (angioplasty and fibrinolysis) are not yet available to a large part of the population in an adequate time (about a quarter of patients with ST-segment AMI arrive at the hospital with more than 6 hours of pain),^19^ the emergence of a new adjuvant therapeutic alternative can have a strong impact on the health of the population. Certainly, many questions still need to be answered: what is the impact of sonothrombolysis on mortality after AMI? Is there a benefit also for patients who underwent only fibrinolysis? Is there a benefit for coronary syndromes without ST-segment elevation? Can sonothrombolysis be started immediately after pain, still in the pre-hospital transport service? This path of knowledge will be long and will take some years. However, the work of Tavares B.,^15^ published in this edition of the *Arquivos* represents an important contribution.
